# Vaginal Cuff Dehiscence Presenting with Free Air 60 Days after Robotic-Assisted Hysterectomy

**DOI:** 10.1155/2017/5052634

**Published:** 2017-10-02

**Authors:** D. Munger, M. Iannamorelli, C. Galvez, C. Service

**Affiliations:** ^1^University of Massachusetts Medical School, 55 N Lake Ave, Worcester, MA 01655, USA; ^2^Berkshire Medical Center Department of Surgery, 725 N Street, Pittsfield, MA 01201, USA; ^3^Berkshire OB/GYN of Berkshire Medical Center, 777 N Street, Pittsfield, MA 01201, USA

## Abstract

**Introduction:**

The vast majority of patients presenting with pneumoperitoneum have visceral organ perforation and require urgent diagnostic laparoscopy. Nonsurgical causes are relatively rare and may be attributed to multiple etiologies.

**Case Presentation:**

Here we describe the case of a 38-year-old Caucasian female who presented to the emergency department with three days of cramping, epigastric abdominal pain. Her physical exam was notable for tenderness to palpation in the epigastric area and abdominal and chest X-rays showed free air under the diaphragm. Free air around the porta hepatis was verified on CT scan. Approximately 90% of pneumoperitoneum cases are due to perforation of visceral organs and therefore require operative management. An urgent exploratory laparoscopy revealed no clear source of free air, but postoperatively the patient developed a large volume of watery discharge from her vagina. Subsequent workup revealed a 1 cm vaginal cuff dehiscence which was later repaired with no postoperative complications.

**Conclusion:**

Although the majority of patients with pneumoperitoneum require urgent exploratory laparoscopy, a careful diagnostic workup may reveal sources of free air that are not related to hollow viscous perforation. Vaginal cuff dehiscence represents a rare yet nonurgent source of pneumoperitoneum. This differential should be considered in light of the possible intra- and postoperative complications of surgery.

## 1. Introduction

Approximately 90% of pneumoperitoneum cases are due to perforation of visceral organs and therefore require operative management [1]. The remaining 10% of cases are considered nonoperative yet they are often still managed with surgical exploration, with one review finding that for 196 case reports describing nonoperative pneumobilia, 45 (27%) patients were brought to the operating room without evidence of visceral perforation [2]. The nonoperative causes are due to a wide range of different sources that can be broadly classified as abdominal, thoracic, gynecologic, and idiopathic [2]. In particular, gynecological causes represent an important source of pneumoperitoneum. This can include sexual intercourse, vaginal douching, knee-chest exercises, pelvic inflammatory disease, and gynecological exam [3]. Here, we illustrate a case of pneumoperitoneum following hysterectomy and postoperative vaginal cuff dehiscence with no clear underlying cause.

## 2. Case Report

A 38-year-old G3P3023 woman presented to the emergency department with three days of cramping epigastric abdominal pain radiating to the periumbilical area. The pain was constant, not associated with food, and accompanied by nausea but without vomiting. She had no fevers or chills and denied gastrointestinal or genitourinary symptoms, including vaginal bleeding or discharge. Her last time of sexual intercourse was 8 days prior to presentation. Her surgical history was significant for an uncomplicated single site robotic-assisted hysterectomy with bilateral salpingectomy and cystoscopy performed 60 days prior, using the* da Vinci*® surgical robot system. This procedure was performed for recurrent cervical intraepithelial neoplasia (CIN) III, a premalignant transformation that typically occurs near the cervical os, which had persisted despite loop electrosurgical excision procedures (LEEP). Prior to presentation, her recovery from this procedure was uncomplicated, with all follow-up appointments followed and only her annual physical exam being scheduled. Her surgical history was also significant for a remote cesarean delivery for oligohydramnios using a Pfannenstiel incision.

**Figure 1:**
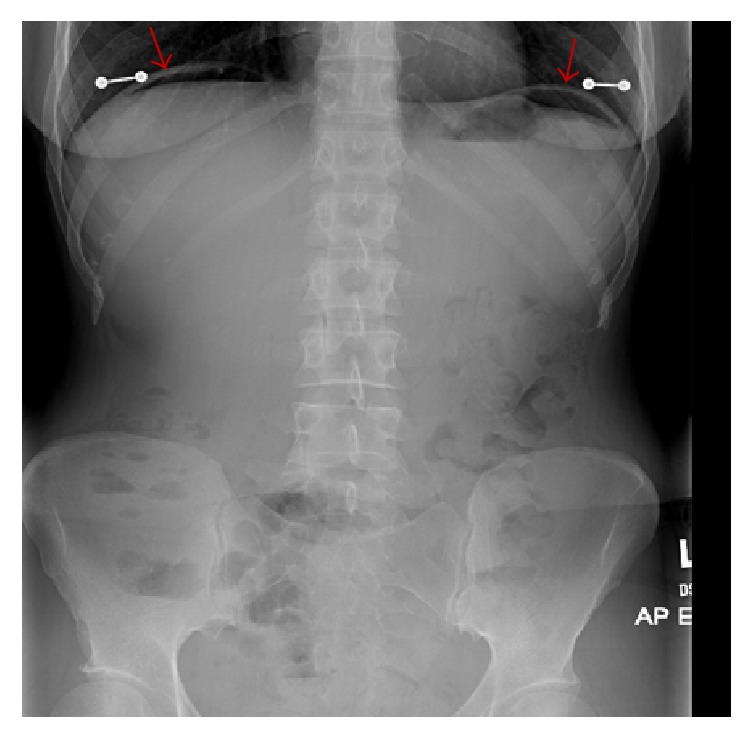
An anterior-posterior (AP) X-ray of the abdomen read as “free intraperitoneal air suggestive of a perforated viscus” (arrows).

**Figure 2:**
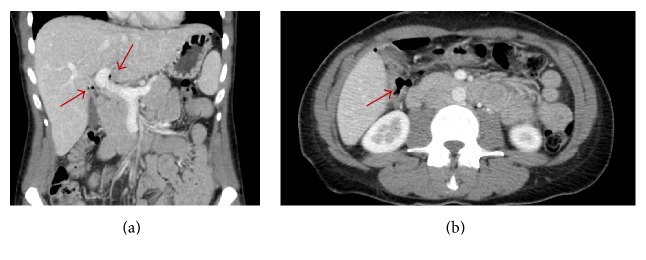
Coronal (a) and transverse (b) views of the CT of the abdomen and pelvis, read as “free intraperitoneal air, findings suggestive of a perforated viscus. There is free air around the porta hepatis which would favor an upper GI source.” Arrows in (a) refer to coronal view of free air around the porta hepatis and in (b) to transverse view of free air around porta hepatis.

On exam her abdomen was soft and nondistended without rebound or guarding, but tender to palpation in the epigastric area. She was afebrile and her vital signs were largely normal with pulse 77, respirations 16, blood pressure 119/68, and oxygen saturation of 97% on room air. A complete blood count showed a white blood cell (WBC) count of 11.8 with 78.4% neutrophils and urinalysis showed cloudy urine with 3+ leukocyte esterase and 11–50 WBCs. X-rays of the chest and abdomen were obtained which showed free air under the diaphragm (Figure 1). A follow-up abdomen and pelvis CT scan without contrast confirmed free air in the abdomen, particularly around the porta hepatis (Figure 2).

Based upon these findings the patient was taken urgently for an exploratory laparoscopy for possible perforated viscus. Intraoperative findings were grossly normal with no intraperitoneal free fluid, edema, inflammatory changes and minimal adhesions which were easily taken down with blunt dissection. The stomach, gallbladder, liver, and pelvis were all visually inspected and the large intestine and small bowel were run. These were found to be normal. An esophagogastroduodenoscopy (EGD) was performed, which demonstrated no ulcerations, blood, or other abnormalities. Warm saline was placed into the intraperitoneal cavity and no gas bubbles were seen following insufflation of the stomach and duodenum in the peritoneal cavity.

The patient tolerated the procedure well and was admitted to the surgical floor for pain control and observation, as well as continued intravenous antibiotics. However, on postoperative day one, the patient stood up and reported a large amount of blood-tinged, watery discharge from her vagina. The Obstetrics and Gynecology Service was consulted as there was a suspicion for vaginal cuff dehiscence. The patient was then taken to the operating room for exam under anesthesia and a 1 cm vaginal cuff dehiscence was found, which was repaired with 0 Monocryl suture. Her postoperative course was uncomplicated, and she completed a 48-hour antibiotic course with eventual resolution of abdominal pain. She was discharged following routine postoperative monitoring.

## 3. Discussion

The case illustrates the importance of investigating gynecological causes as a source of pneumoperitoneum. Numerous case reports have demonstrated pneumoperitoneum following hysterectomy and sexual intercourse. However, a review of the literature reveals that cases of pneumoperitoneum following both genital and oral-genital intercourse are typically associated with onset of symptoms 2–4 hours after sexual activity [3–5]. Similarly, the longest timespan for pneumoperitoneum associated with laparoscopic hysterectomy previously reported was 48 days postoperatively with no preceding sexual intercourse [6].

Here we present a case of pneumoperitoneum following vaginal cuff dehiscence 60 days after robotic-assisted hysterectomy-bilateral salpingectomy, with the most recent episode of sexual intercourse being 8 days prior. Therefore, while both of these are gynecological risk factors for dehiscence, neither is temporally related to this patient's symptoms. This case illustrates the importance of considering these causes of pneumoperitoneum in patients with a relevant gynecological history despite a timeframe that, based on published literature, is not typically associated with this clinical presentation. Such careful investigation could prevent the need for exploratory surgery should a gynecological workup reveal another possible source of air infiltration into the abdomen.

This is relevant because surgical intervention for both diagnostic and therapeutic purposes carries a number of rare yet well-established risks. Possible iatrogenic sources of morbidity and mortality include intraoperative complications from surgical manipulations, adverse reactions to anesthesia agents, medication errors, and postoperative infections. Although these risks are low, they illustrate that even a procedure as straightforward as a diagnostic laparoscopy is not entirely benign.

Furthermore, abdominal surgery is associated with long-term consequences of intra-abdominal adhesions which can lead to complications such as small-bowel obstruction, pain, and female infertility [7]. Complications from adhesions have been reported to occur in approximately 34.6% of patients who underwent abdominal surgery and as long as 50 years following surgery [8, 9]. In women, intra-abdominal adhesions are responsible for 20–40% of female secondary infertility [9].

Although this case describes a patient who presented with a potentially acute condition, it highlights the need to balance both short- and long-term risks of operative management against the possible immediate morbidity and mortality of conservatively treated pneumoperitoneum. Although such clinical decision-making is both challenging and often based upon incomplete information, it lies at the core of physician's responsibility to “*first, do no harm*.”

## 4. Conclusion

Free air in the abdomen following months-prior gynecological surgery and/or sexual intercourse represents a relatively rare yet nonurgent clinical picture when compared with the majority of patients who present with this condition. Most cases of pneumoperitoneum are due to hollow viscous perforation, and therefore require operative management. Although urgent exploratory laparoscopy is the standard of care for most patients with pneumoperitoneum, these alternate causes should be taken into consideration when weighing whether a patient would benefit from surgical intervention.
